# Supplementary material for: Hybrid Models and Biological Model Reduction with PyDSTool
Source: PLoS Comput Biol. 2012 Aug 9;8(8):e1002628. doi: 10.1371/journal.pcbi.1002628 (PMC3415397; doi:10.1371/journal.pcbi.1002628)
Supplement: Text S4 — Complete source code for the PyDSTool package (version 0.88.120504). Includes API documentation and help files linking to web pages. This file is identical to the current public release on Sourceforge.net. (ZIP) [file pcbi.1002628.s004.zip › PyDSTool/html/PyDSTool.MProject.binary_feature-class.html]

xml version="1.0" encoding="ascii"?


PyDSTool.MProject.binary\_feature


| Home | Trees | Indices | Help | PyDSTool |
| --- | --- | --- | --- | --- |

Package PyDSTool ::
Module MProject ::
Class binary\_feature

[hide private]

[frames] | no frames]

# Class binary\_feature

source code

```
object --+            
         |            
   feature --+        
             |        
  feature_leaf --+    
                 |    
   ql_feature_leaf --+
                     |
                    binary_feature
```

---

Use this as a binary switch feature, toggled by a given variable name
'varname' that is supplied in the pars dict at initialization.


|  |  |
| --- | --- |
| Instance Methods | [hide private] |

|  |  |
| --- | --- |
| evaluate(self, target) | source code |

|  |  |
| --- | --- |
| \_find\_idx(self)  Internal function for finding index in trajectory meshpoints at which containment first failed. | source code |

**Inherited from `feature_leaf`**:
`__repr__`,
`__str__`,
`set_ref_traj`

**Inherited from `feature_leaf`** (private):
`_residual_info`

**Inherited from `feature`**:
`__call__`,
`__eq__`,
`__hash__`,
`__init__`,
`__ne__`,
`finish`,
`info`,
`postprocess_ref_traj`,
`prepare`,
`reset_metric`,
`validate`

**Inherited from `object`**:
`__delattr__`,
`__getattribute__`,
`__new__`,
`__reduce__`,
`__reduce_ex__`,
`__setattr__`


|  |  |
| --- | --- |
| Properties | [hide private] |

**Inherited from `object`**:
`__class__`


|  |  |
| --- | --- |
| Method Details | [hide private] |

|  |  |
| --- | --- |
| evaluate(self, target) | source code |

Overrides: feature.evaluate

|  |  |
| --- | --- |
| \_find\_idx(self) | source code |

Internal function for finding index in trajectory meshpoints at which
containment first failed. Defaults to returning None and must be
overridden by a class that has access to a mesh.

Overrides: feature.\_find\_idx: *(inherited documentation)*

  


| Home | Trees | Indices | Help | PyDSTool |
| --- | --- | --- | --- | --- |

|  |  |
| --- | --- |
| Generated by Epydoc 3.0.1 on Fri May 4 15:24:06 2012 | http://epydoc.sourceforge.net |
